# First Report on Abnormal Renal Function in Acute Hepatitis E Genotype 1 Infection

**DOI:** 10.3390/pathogens12050687

**Published:** 2023-05-08

**Authors:** Amal A. Elkhawaga, Mohamed A. El-Mokhtar, Amal A. Mahmoud, Wael Esmat Ali, Doaa Safwat Mohamed, Ayat M. Kamel, Ahmed Atef Mesalam, Nermien H. S. Mousa, Ahmed M. Ashmawy, Essam M. Abdel Aziz, Ibrahim M. Sayed, Haidi Karam-Allah Ramadan, Yasmine Samy Elkholy

**Affiliations:** 1 Department of Medical Microbiology and Immunology, Faculty of Medicine, Assiut University, Assiut 71515, Egypt; 2 Department of Clinical Pathology, Faculty of Medicine, Assiut University, Assiut 71515, Egypt; 3 Department of Clinical Pathology, Faculty of Medicine, Al-Azhar University Assuit Branch, Assiut 71524, Egypt; 4 Department of Microbiology & Immunology, Faculty of Pharmacy, Sohag University, Sohag 82524, Egypt; 5 Microbiology and Immunology Department, Faculty of Pharmacy, Assiut University, Assiut 71515, Egypt; 6 Department of Therapeutic Chemistry, Pharmaceutical and Drug Industries Research Institute, National Research Centre (NRC), Dokki, Cairo 12622, Egypt; 7 Botany & Microbiology Department, Faculty of Science, Assiut University, Assiut 71515, Egypt; 8 Department of Internal Medicine, Gastroenterology and Hepatology Unit, Faculty of Medicine, Assiut University, Assiut 71515, Egypt; 9 Department of Internal Medicine, Nephrology Division, Faculty of Medicine, Assiut University, Assiut 71515, Egypt; 10 Department of Tropical Medicine and Gastroenterology, Faculty of Medicine, Assiut University, Assiut 71515, Egypt; 11 Department of Medical Microbiology and Immunology, Faculty of Medicine, Cairo University, Cairo 12613, Egypt

**Keywords:** HEV, kidney function test, acute infection, genotype 1, recovery, renal disorders

## Abstract

Impaired renal functions have been reported with Hepatitis E virus (HEV) infections, especially with genotypes 3 and 4. These complications were reported during the acute and chronic phases of infection. HEV genotype 1 causes acute infection, and the effect of HEV-1 infections on renal functions is not known. We examined the kidney function parameters in the serum of HEV-1 patients (AHE, n = 31) during the acute phase of infection. All of the included patients developed an acute self-limiting course of infection, without progression to fulminant hepatic failure. We compared the demographic, laboratory, and clinical data between AHE patients with normal kidney function parameters and those with abnormal renal parameters. Out of 31 AHE patients, 5 (16%) had abnormal kidney function tests (KFTs) during the acute phase of infection. Three patients had abnormal serum urea and creatinine, and two patients had either abnormal urea or creatinine. Four out of five patients had an estimated glomerular filtration rate (eGFR) below 60 mL/min/1.73 m^2^. AHE patients with abnormal KFTs were older and had a lower level of albumin, but a slightly elevated alanine transaminase (ALT) compared to AHE patients with normal KFTs. There were no significant differences between the two groups in terms of age, sex, liver transaminase levels, and the viral load. Similarly, the clinical presentations were comparable in both groups. Interestingly, these KFTs in patients with abnormal renal parameters returned to normal levels at the recovery. The serum creatinine level was not correlated with patients’ age or liver transaminase levels, but it was significantly negatively correlated with albumin level. In conclusion, this study is the first report that evaluated KFTs in patients during the acute phase of HEV-1 infections. Impaired KFTs in some AHE patients resolved at convalescence. KFTs and renal complications should be monitored during HEV-1 infections.

## 1. Introduction

The Hepatitis E virus is an RNA virus, and the isolates that infect humans belong to the genus *Paslahepevirus,* subfamily *Orthohepevirinae,* and family *Hepeviridae* [1]. The viral genome includes three open reading frames (ORFs) that are required for virus replication inside the host (ORF1), synthesis of virus capsid and assembly (ORF2), and virus release (ORF3) [2]. The genus *Paslahepevirus* includes eight genotypes, and three of them are not confirmed as human pathogens (HEV-5, HEV-6, and HEV-8) [1]. Four genotypes (HEV-1–HEV-4) cause acute hepatitis in humans. In addition, HEV-3 and HEV-4 cause chronic infections [3,4,5,6]. No confirmed cases of chronic infection have been reported with HEV-1 or HEV-2 [6]. HEV-1 is common in Africa and India and the fecal–oral route is the main route of transmission [6]. HEV-3 is endemic in Europe where pigs are the main reservoir and source of infections [7]. HEV-4 is mainly distributed in China and to a lesser extent in Europe, and the virus crosses several species [8,9,10]. HEV-3 and HEV-4 are mainly transmitted through eating undercooked infected liver, sausages, and meat pieces [11,12]. There are other methods for HEV transmission such as the transfusion of HEV-contaminated blood or blood products including red blood cells, platelets, and fresh frozen plasma [13]. HEV infection develops in recipients of these contaminated bloods/products, especially if those recipients have attenuated immunity due to immunosuppressive therapies for organ transplants or because of hematological malignancy [14]. HEV-3 is the most reported genotype associated with HEV transfusion-transmitted hepatitis and, less reported with HEV-4 [14]. In addition, HEV can be transmitted during pregnancy. The outcomes of HEV infection in the mother and offspring depend on the transmitting genotypes and the trimester of pregnancy [15,16]. HEV infection in pregnant women can lead to fulminant hepatic failure, especially if the infection develops in the third trimester of pregnancy [17,18,19]. Vertical transmission of HEV is common, and severe fetal complications have been reported such as preterm delivery and/or perinatal morbidity and mortality [17,18,19].

The liver is the main target for HEV infection. However, the replication and pathogenesis of HEV in organs other than the liver have been documented, especially with the isolates that cause chronic infections [3,5]. These extrahepatic disorders include renal or neurological manifestations, cryoglobulinemia, and pancreatitis, which are mediated by either the virus pathogenicity in these tissues or the deposition of immunocomplexes [5]. Renal disorders reported in HEV-infected patients include glomerulonephritis, an impaired glomerular filtration rate, and mixed cryoglobulinemia [5,20,21]. Guillain-Barré syndrome, Bell’s palsy, neuropathy, and myositis are examples of neurological manifestations reported during HEV infection [5,14]. In addition, thrombocytopenia, aplastic anemia, and hemolytic anemia were also reported in HEV-infected patients [5,14]. Most of the previously mentioned extrahepatic disorders were reported during HEV-3 or HEV-4 chronic infections. However, limited data are available on the link between HEV-1 and extrahepatic manifestations. Several studies have reported the pathogenesis of HEV in the kidney. HEV-3 is the most reported strain with glomerular disorders. Acute and chronic infections mediated by HEV-3 are associated with acute kidney injury (AKI) and a reduced glomerular filtration rate (GFR) [22,23]. Kidney functions are restored upon viral clearance [22,23]. HEV-4 infections are also associated with acute and chronic kidney diseases [24,25], and HEV-4-associated chronic kidney disease is a prognostic factor for HEV-associated mortality rate [25]. The association between HEV-1 and kidney abnormalities is not completely known. Most of the known data were derived from experimental animal models and/or in vitro cell culture models. HEV-1 infection induced histopathological changes in the kidney of infected Z:ZCLA Mongolian gerbils [26]. HEV-1 replicates in primary proximal epithelial cells, and HEV RNA and HEV protein increase over time; the infection activates the inflammatory cytokines IL-8 and IL-6, but not chemokine transcripts or kidney injury markers [27]. In India, where HEV-1 is a common genotype, several HEV-infected cases were found to be complicated by renal failure or cholemic nephrosis [28,29]. In Egypt, HEV-1 is the most commonly reported strain associated with acute hepatitis and fulminant hepatic failure [30]. Though the research on HEV infection is growing in Egypt, no data regarding KFTs during acute self-limiting HEV-1 infections have been reported.

Herein, we aimed to investigate the impact of HEV-1 infections on renal parameters during the acute phase of infection. We assessed the KFTs in the sera of patients with acute hepatitis E (AHE) infections who were infected with HEV-1, and compared the demographic and laboratory data between patients with normal KFTs and patients with abnormal KFTs.

## 2. Materials and Methods

(I) Patients: This study included an analysis of acute hepatitis E patients (AHE, n = 31) admitted to outpatient clinics and Assiut University Hospitals, Egypt, during the period from 2020 to 2023. All patients were symptomatic and with clinical manifestations of acute hepatitis [30,31]. Blood samples were collected from the patients on admission. The included samples tested negative for other hepatotropic viruses (hepatitis A virus (HAV), hepatitis B virus (HBV), hepatitis C virus (HCV), cytomegalovirus (CMV), Epstein Barr virus (EBV), and adenovirus). Patients who tested positive for one or more autoimmune markers, and patients with suspected drug-induced liver injury were excluded from the study, as described before [31,32]. Patients with co-morbidities such as diabetes mellitus, ischemic heart disease, hypertension, or chronic kidney diseases or abnormal renal imaging by ultrasound were excluded. Causes of abnormal KFTs were excluded such as hypotension, septicemia, drugs, or obstructive uropathy. HEV diagnosis was performed using serological and molecular approaches. The serological methods included the detection of anti-HEV IgM and anti-HEV IgG by commercial ELISA assays (AB Diagnostic Systems GmbH, Berlin, Germany). The molecular method was performed by RT-qPCR for the amplification and quantification of HEV RNA using primers and probes targeting HEV ORF2/3 regions; the following primers were used: forward primer: 5′-GGTGGTTTCTGGGGTGAC-3, reverse primer: 5′-AGGGGTTGGTTGGATGAA-3, and probe: 5′FAM-TGATTCTCAGCCCTTCGC-3′TAMRA [30,31,33]. A nested PCR assay was used to amplify HEV ORF2 and confirm the genotyping [34,35]. Informed consent was obtained from the subjects involved in the study. This protocol was approved by the Institutional Review Board (IRB no 17300294) at the Faculty of Medicine, Assiut University, Egypt, in accordance with the provisions of the Declaration of Helsinki.

(II) Liver function and KFTs: The liver function and KFTs were analyzed in patients’ samples using a clinical chemistry analyzer (Mindray BS-230) according to the manufacturer’s instructions. KFTs included serum urea, creatinine, and eGFR. The eGFR is calculated based on MDRD 4-Variable Equation [36,37]. The calculation was based on four parameters: age, gender, serum creatinine, and race.

Normal values of these laboratory markers: serum urea 15–45 mg/dL, serum creatinine 0.7–1.3 mg/dL in males and 0.6–1.1 mg/dL in females, serum albumin 3.5–5.2 g/dL, total bilirubin 0.1 to 1.2 mg/dL, direct bilirubin less than 0.3 mg/dL, ALT 0–45 IU/L, aspartate transaminase (AST) 0–34 IU/L, alkaline phosphatase (ALP) 45–150 IU/L.

(III) Statistics: Statistical analyses were carried out using GraphPad Prism 9. The data were expressed as mean ± standard deviation (SD) or median (interquartile range (IQR)) and/or the range (minimum–maximum). They were compared using a Student’s *t*-test, ANOVA, or Mann–Whitney U-test. All tests were two-tailed and the *p*-values < 0.05 were considered significant. The correlation between serum creatinine and other parameters was analyzed using the Spearman’s correlation test.

## 3. Results

### 3.1. Assessment of KFTs in AHE Patients

The study includes serum samples of the AHE patients (n = 31) and their kidney function parameters during the acute phase of infection. All of the included patients had acute self-limiting disease, with no progression to fulminant hepatic failure. The demographic and laboratory parameters for these patients/samples are presented in Table 1. The median age was 43 years, and almost half of the patients were male. All patients had elevated liver transaminases, alkaline phosphatase, and bilirubin levels as signs of acute hepatitis. The mean levels of ALT and AST were 503 IU/L and 335 IU/L, respectively. The mean levels of plasma urea, creatinine, and GFR were 32 mg/dL, 0.97 mg/dL, and 93.65 mL/min/1.73 m^2^, respectively. The mean albumin level was 3.2 g/dL.

Five out of 31 patients (16.12%) had elevated serum creatinine and urea levels and impaired GFR during the acute phase of infection (Table 2). Three patients (Pt# 1, 2, and 5) had abnormal sera urea (>45 mg/dL) and creatinine (>1.1 mg/dL) levels. These patients also had impaired GFR (<60 mL/min/1.73 m^2^). The fourth patient (patient #3) had an elevated urea level, but a normal creatinine level and the GFR (>60 mL/min/1.73 m^2^) was not impaired. The fifth patient (patient #4) had normal urea, but elevated creatinine levels and reduced GFR (Table 2). Of those AHE patients with abnormal KFTs were three males and two females, and their ages ranged from 36 to 70 years. In general, the patients, except patient #4, had hypoalbuminemia. The clinical presentations of acute hepatitis were typical. All patients developed jaundice and none of them had bleeding or encephalopathy. Other symptoms such as fever, abdominal pain, and vomiting were variable. Vomiting was reported by three out of five patients (pt #1, pt #4, and pt #5), while fever (pt #2 and pt #3) and abdominal pain (pt #1 and pt #4) were reported by two out of five patients.

### 3.2. Comparison between AHE patients with Normal and Abnormal KFTs

Then, we compared the demographic, clinical, and laboratory parameters between AHE patients with normal KFTs (n = 26) and those with abnormal KFTs (n = 5). There were no significant differences between either group in terms of age, gender, LFTs, albumin level, and viremia (Table 3). The AHE patients with abnormal KFTs were older and had lower blood albumin compared to those with normal KFTs. Regarding LFTs, AHE patients with abnormal KFTs had higher ALT levels, but lower AST and direct bilirubin levels compared to those with normal KFTs (*p* > 0.05). Regarding the clinical manifestations, the symptoms were comparable in both groups. Jaundice developed in 100% of patients in both groups, followed by vomiting (50% vs. 60% in acute HEV with normal and abnormal KFTs, respectively).

### 3.3. Assessment of KFTs at Recovery

**Figure 1:**
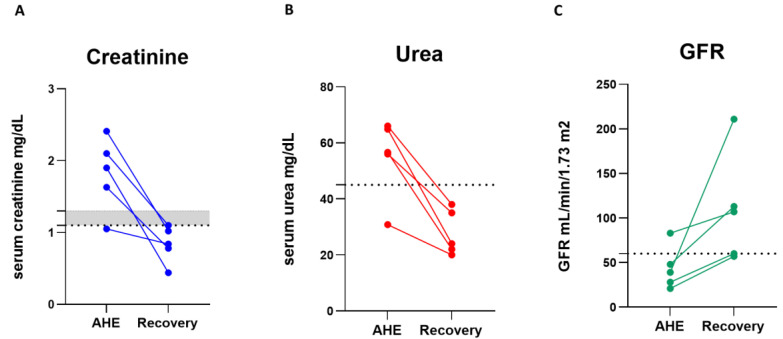
Results of KFTs (creatine (**A**), urea (**B**), and GFR (**C**)) of AHE patients with abnormal renal parameters during recovery. Dashed lines represent the values that separate normal and abnormal renal parameters (upper normal value level).

The results of AHE patients with abnormal KFTs at convalescence showed that none of these patients developed further renal complications and the urea and creatinine levels returned to normal values (Figure 1). The GFR was improved in all patients. The improvement in KFTs was concurrently matched with the absence of viremia and the disappearance of clinical symptoms.

### 3.4. Correlation between Serum Creatinine and Other Laboratory and Demographic Parameters

Then, we assessed the correlation between serum creatinine and other laboratory and demographic parameters in AHE patients to identify any possible risk factors associated with elevated serum creatinine or impaired GFR in these patients. We found that serum creatinine was significantly negatively correlated with serum albumin (Spearman r= −0.4042, 95% confidence interval: −0.6696 to −0.04725, and *p*-value 0.0241) and significantly positively correlated with serum urea (Spearman r= 0.5814, 95% confidence interval: 0.2758 to 0.7802, and *p*-value 0.0006). The serum creatinine did not significantly correlate with age, ALT, AST, ALP, or bilirubin levels (Table 4).

## 4. Discussion

HEV replicates mainly in the liver and in other organs beyond the liver such as the kidney, neurons, and GIT. [5,38]. The disorders that developed in HEV-infected patients outside the liver became a feature of HEV infection. Although the research on HEV extrahepatic manifestations is increasing, a complete understanding of HEV pathogenesis in these organs has not been achieved. Among these organs is the kidney, and renal disorders are reported by several studies with HEV infections. Kamar et al. reported that HEV infections, either acute or chronic, were associated with kidney dysfunction [23]. The analysis of kidney biopsies of organ transplant patients revealed that several renal disorders were recorded, such as IgA nephropathy and glomerulonephritis [23]. The renal functions returned to normal after viral clearance [23]. Several other reports showed that HEV is excreted in the urine of HEV-3 and/or HEV-4-infected patients or non-human primates with evidence of kidney injury [24,39]. The replication of HEV in the kidney was confirmed by the detection of negative-strand HEV RNA, HEV ORF2 antigen, and other histopathological changes in known animal models such as pigs, non-human primates, rabbits, and Mongolian gerbils [24,26,40,41,42]. HEV is released in the urine, and the urine of HEV-infected monkeys was found to contain viral particles that were infectious to naïve monkeys [24]. The inoculated monkey developed acute hepatitis and HEV RNA was detected in the serum, stool, and urine [24]. HEV protein has also been recorded in the urine of infected patients and animal models [24,39]. A higher level of viral protein (ORF2 Ag) compared to HEV nucleic acid was detected in the urine than in the stool or serum of the same animal/or patient [24,39]. Acute renal failure was also reported with acute HEV infections [43,44]. Most of the HEV infections associated with renal disorders were reported with HEV-3 and HEV-4 [5,24,45,46]. Regarding HEV-1 infection, the link between viral infection and acute kidney injury (AKI) and/or KFTs is not clear. Karki et al. reported a case of acute HEV infection associated with hemolysis and kidney injury, which required renal dialysis [28]. Nayak et al. reported a case of acute HEV infection that was complicated by cholemic nephrosis (bile cast nephropathy), which could lead to AKI [29]. Although the viral genotype was not reported in the previous studies, HEV-1 is the most common circulating strain in India [47]. In the previous reports, acute HEV infections were complicated by end-stage renal diseases or other complications. However, the KFTs during acute self-limiting disease caused by HEV-1 are not known. Here, we assessed the kidney function parameters in HEV-1 infections during the acute phase of infections that were not complicated by end-stage renal diseases. All patients enrolled in this study developed acute self-limiting disease. We excluded patients who developed fulminant hepatitis, since those patients could develop renal abnormalities that are a consequence of hepatic failure.

In this study, we assessed the KFTs (mainly serum creatinine and urea levels and estimated GFR) during the acute phase of HEV-1 infections. In total, 5 out of 31 patients (16%) showed abnormal KFTs that returned to normal values during the recovery. None of these patients had a history of previous kidney diseases or medication history that could be associated with renal abnormalities. The findings suggest that poor kidney function was associated with the infection. Similarly, Brehm and colleagues reported that 10 out of 69 (14.5%) acute HEV-infected patients (mainly caused by HEV-3) had higher serum creatinine during the acute phase of infection [22]. Interestingly, five out of the previous ten patients had specifically increased creatinine levels during infection, but not before infection, suggesting that the increase in the creatinine level was only associated with HEV infection [22]. Moreover, in the present study, the creatinine and urea levels returned to normal values and the GFR was improved after HEV clearance and the disappearance of clinical symptoms. Similar findings were also reported by Brehm et al. during acute HEV-3 infection [22]. On the other hand, Marion et al. showed that the acute phase of HEV-3 infection was not associated with impaired renal functions [39]. The discrepancy between the findings of Brehm et al. and Marion et al., despite dealing with the same viral genotype, regarding the impact of HEV infection on renal function could be attributed to the characteristics of the enrolled patients. Brehm et al. focused on immunocompetent patients [22], while the study conducted by Marion et al. was limited to immunocompromised patients (solid organ transplants) who received immunosuppressive medication following the transplantation [39]. In this study, none of the patients were organ transplant recipients, nor had they received immunosuppressive drugs. This could explain the similarity of this study’s findings with the findings reported by Brehm and colleagues [22].

No difference was observed in the current study regarding age, gender, liver transaminases, serum albumin, and bilirubin between AHE patients with normal KFTs and those with abnormal KFTs. Although not statistically significant, the AHE patients with normal KFTs had slightly higher serum albumin, AST, and direct bilirubin levels, while the AHE patients with abnormal KFTs were older. These findings require further confirmation in future studies. As the KFTs returned to normal values at recovery, we excluded the supposition that age was the causative factor of the abnormal KFTs. A previous study showed that AHE patients were older and had lower liver transaminases and bilirubin levels compared to those with acute hepatitis A (AHA) infections [22]. On the other hand, none of the AHA patients had abnormal KFTs [22]. In addition, the results of this study are comparable with those recently reported by Fan et al., who compared symptomatic HEV-4 infection with and without chronic kidney diseases [25]. The symptomatic HEV-4 patients with kidney diseases were older and had lower albumin levels and higher bilirubin and creatinine levels than the HEV-infected patients without kidney disease [25].

Regarding the viral load, the viremia was comparable between the AHE patients with normal KFTs and those with abnormal KFTs, suggesting that the HEV load did not impact the development of poor kidney function. Parallelly, Brehm and colleagues showed that there was no correlation between serum creatinine and HEV load [22]. In immunocompromised HEV-infected patients, there were no differences in GFR or serum creatinine between patients who tested positive for HEV RNA in urine and patients who tested negative for HEV RNA in urine [39]. HEV extrahepatic manifestations are caused by either the direct viral replication or host immune responses against the virus that are associated with the deposition of the immune complexes in different organs [5]. Since the viral load was not associated with abnormal KFTs, the renal abnormalities could be caused by immune-mediated mechanisms. However, we could not exclude the replication of the virus in the kidney as another cause. Further studies should ascertain this point.

Our previous study showed that HEV-1 could replicate in the proximal tubules (PT) epithelium; however, the replication was not associated with the activation of all inflammatory chemokines nor transcripts of AKI such as IL-18, Kidney Injury Molecule 1 (KIM-1), and neutrophil gelatinase-associated lipocalin (NGAL) [27]. However, when the PT epithelium was cocultured with PBMCs and challenged with HEV-1, there was a significant increase in the transcript levels of inflammatory and AKI markers [27]. Similarly, the infection of Mongolian gerbils with a stool-derived HEV-1 virus showed an accumulation of inflammatory immune cells in the liver, kidney, and spleen [26]. Collectively, these findings and our findings suggest that HEV-1 could replicate in the kidney and induce an immune response.

Although this study could not show any predicative factor for the development of renal abnormalities in AHE patients (probably due to the small sample size), it highlights that the KFTs should be monitored during acute HEV infection. KFTs are performed on the blood samples in a similar manner as LFTs. Therefore, this study recommends the inclusion of KFTs with LFTs during the analysis of acute HEV infections, especially in old age. This could reduce the risk of renal complications that might develop during the infection. The cost of KFTs is low and the performance is simple.

This study has some limitations. The available analyzed samples were small in number. We could not obtain urine samples from the AHE patients, which impeded the monitoring of HEV markers and albumin in urine. A recent study showed that HEV Ag was taken by the renal cells and excreted in urine more than 10-fold than in plasma [48]. Future studies should analyze HEV in the urine of HEV-infected patients.

## 5. Conclusions

This study is the first report that showed KFTs in patients with acute HEV-1 infections. Impaired KFTs were observed in some AHE patients that resolved at convalescence. Future studies should be conducted to assess the complications associated with abnormal KFTs during acute HEV-1 infections.

## Figures and Tables

**Table 1:** Characteristics of acute HEV patients enrolled in the study.

Items	Acute HEV (n = 31)
Age (years) (Median, IQR)	43 (24–52)
Sex (M/F)	16/15 (51.6%/48.4%)
Serum urea (mg/dL) Median (Range)	28.12 (15.10–66)
Serum creatinine (mg/dL) Median (Range)	0.77 (0.64–2.41)
GFR (mL/min/1.73 m^2^) Median (Range)	94 (21–154)
Liver function tests (LFTs) (Median, IQR)	
ALT (IU/L)	290 (180–631)
AST(IU/L)	233 (134–396)
ALP (IU/L)	149 (124–200)
Total bilirubin (mg/dL)	8.50 (4.48–11.87)
Direct bilirubin (mg/dL)	6.43 (3.80–8.91)
Serum albumin g/dL Median (Range)	3.10 (1.50–4.40)

**Table 2:** Demographic, laboratory, and clinical criteria of AHE patients with abnormal KFTs.

	Patient 1	Patient 2	Patient 3	Patient 4	Patient 5
**Age**	48	70	41	57	36
**Sex**	Male	Female	Male	Male	Female
**Kidney function test**					
Urea (mg/dL)	56.60	64.90	56	30.80	66
Creatinine (mg/dL)	1.63	2.41	1.05	1.90	2.10
GFR (mL/min/1.73 m^2^)	48	21	above 60	39	28
**LFTs**					
ALT (IU/L)	201	399	488	91	572
AST (IU/L)	154.60	203	144	108	344
ALP (IU/L)	114	61	159	165	143
Total bilirubin (mg/dL)	8.36	10.05	6.92	10.58	3.80
Direct bilirubin (mg/dL)	5.11	7.50	5.81	4.09	3.10
Serum Albumin g/dL	1.50	2.90	2.20	3.70	3
**Clinical presentations**					
Jaundice	Yes	Yes	Yes	Yes	Yes
Fever	No	Yes	Yes	No	No
Vomiting	Yes	No	No	Yes	Yes
Diarrhea	No	No	Yes	No	No
Abdominal pain	Yes	No	No	Yes	No
Bleeding	No	No	No	No	No
Encephalopathy	No	No	No	No	No

**Table 3:** Characteristics of acute HEV patients with normal and abnormal KFTs.

	Acute HEV with Normal KFTs (n = 26)	Acute HEV with Abnormal KFTs (n = 5)	Statistics (*p*-Value)
Age (years) (mean ± SD)	39.73± 14.82	50.40± 13.50	ns
Sex (M/F)	13/13 (50%: 50%)	3/2 (60%: 40%)	ns
Mean viral load (IU/mL)	1.11 × 10^4^	1.37 × 10^4^	ns
**LFTs** (Median-IQR)			
ALT (IU/L)	283 (175.30–715.30)	399 (146–530)	ns
AST(IU/L)	257.90 (129.80–462)	154.60 (126–273.50)	ns
ALP (IU/L)	149.50 (129.30–201)	143 (87.50–162)	ns
Total bilirubin (mg/dL)	8.81 (4.47–13.78)	8.36 (5.36–10.32)	ns
Direct bilirubin (mg/dL)	6.77 (3.69–9.21)	5.11 (3.60–6.66)	ns
Serum albumin g/dL	3.29 ± 0.76	2.66± 0.84	
(mean ± SD)			ns
**Clinical manifestations**			
Jaundice	26/26 (100%)	5/5 (100%)	ns
Fever	12/26 (46%)	2/5 (40%)	ns
Vomiting	13/26 (50%)	3/5 (60%)	ns
Diarrhea	7/26 (26.9%)	1/5 (20%)	ns
Abdominal pain	12/26 (46%)	2/5 (40%)	ns
Bleeding	0/26 (0%)	0/5 (0%)	ns
Encephalopathy	0/26 (0%)	0/5 (0%)	ns

Ns: non-significant, *p*-value more than 0.05.

**Table 4:** Correlation between serum creatinine and other parameters.

Variable	Result
Age	*r*-value: 0.2774
	*p*-value: 0.1308
**LFTs**	
ALT	*r*-value: −0.01210
	*p*-value: 0.9485
AST	*r*-value: 0.05225
	*p*-value: 0.7801
ALP	*r*-value: −0.2229
	*p*-value: 0.2282
Total bilirubin	*r*-value: 0.1569
	*p*-value: 0.3991
Albumin	*r*-value: −0.4042
	*p*-value: 0.0241 *
**KFTs** **Urea**	*r*-value: 0.5814
	*p*-value: 0.0006 ***

*, *** mean *p* value < 0.05, 0.001, respectively.

## Data Availability

The study data are present in the main text and for further inquiries, please contact the corresponding authors.
